# Multiple Meniscus Depinning Transitions in Open Capillary Slits

**DOI:** 10.1021/acs.jpcb.5c07472

**Published:** 2025-12-15

**Authors:** Jiří Janek, Andrew O. Parry, Alexandr Malijevský

**Affiliations:** † Research Group of Molecular and Mesoscopic Modelling, The Czech Academy of Sciences, Institute of Chemical Process Fundamentals, Prague 165 02, Czech Republic; ‡ Department of Mathematics, 4615 Imperial College London, London SW7 2BZ, U.K.; § Department of Physical Chemistry, 52735 University of Chemistry and Technology Prague, Prague 166 28, Czech Republic

## Abstract

We study edge-induced meniscus depinning transitions in confined fluids using a combination of macroscopic theory and classical density functional theory (DFT). The study focuses on macroscopically long slit geometries of width *L* bounded by planar walls where the open end has an overlap *D*, where sharp edges introduce distinct meniscus morphologies and continuous depinning transitions. The macroscopic analysis predicts four distinct condensed states: fully pinned, partially pinned, and depinned whose stability depends sensitively on the wall separation ratio *D*/*L* and the Young contact angle θ. These transitions are second order, if the walls are partially wet, and third order if they are complete wet (θ = 0) or completely dry (θ = π). Microscopic DFT calculations confirm the existence and sequence of these morphologies in very good quantitative agreement with the macroscopic predictions, except for complete wetting, where the presence of thick adsorbed films effectively reduces the accessible slit width.

## Graphical abstract

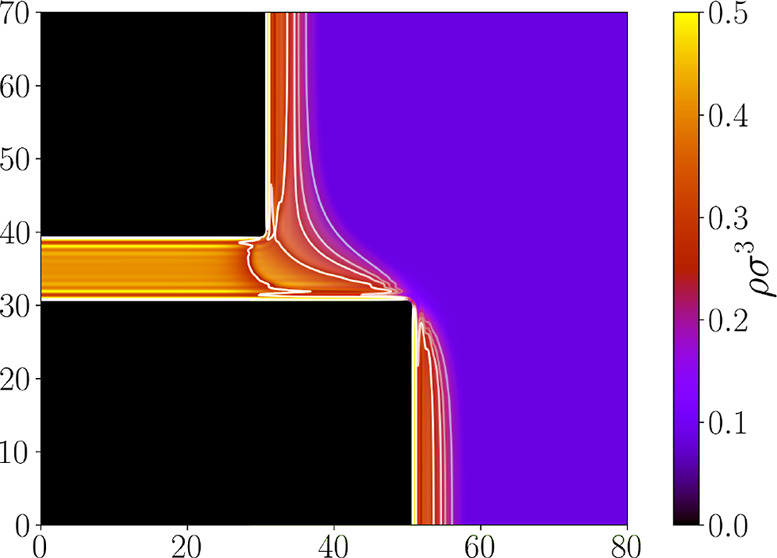

## 1 Introduction

Fluids at interfaces, and in confined geometries, display a wealth of behavior, even at equilibrium, which are not present in bulk systems.^1–8^ Among these are several phase transitions which are induced by the interaction with the walls of the confining geometry. Perhaps the most notable and well-studied among these are wetting transitions at planar walls^1–4,8,11,12^ associated with the vanishing of the contact angle, and capillary condensation when a fluid confined in a capillary slit of width *L*, condenses at a chemical potential μ_cc_(*L*) = μ_sat_–δμ_cc_, below that of bulk saturation μ_sat_. The location of this condensation is described by the macroscopic Kelvin equation^9,10^

1 $$\delta \mu_{cc}=\frac{2\gamma \;\cos\theta }{L\Delta \rho }$$

whose accuracy, particularly for partial wetting, has been tested extensively using microscopic density functional methods. Here, γ is the liquid–gas surface tension and Δρ is the density difference of the coexisting bulk liquid and gas phases. Studies of wetting on planar walls, and of condensation in capillary slit geometries, are often seen as useful idealizations of wetting and adsorption on rough surfaces and in porous materials. While this is undoubtedly true, what is perhaps not widely appreciated is that, further phase transitions occur when these idealized geometries are even slightly altered–in particular when the confinement leads to the formation of an interface separating different fluid phases. This occurs for example when a capillary slit is capped at one end, but open at the other, in which case the condensed liquid-like phase necessarily has a meniscus.^13–23^ The manner in which this meniscus unbinds from the capped end of the capillary then determines the order of the capillary condensation transition, which may be continuous or first-order. Other examples include wedge filling transitions for fluids adsorbed in wedges made from two planar walls that meet at a given opening angle.^24–29^ The order of these transitions depends sensitively on the range of the microscopic intermolecular forces and is different to that for wetting at planar walls. As a final example we mention adsorption near a completely wet surface which is smooth but locally concave, which leads to the phenomenon of meniscus osculation–a higher-order phase transition associated with the formation of a macroscopic meniscus when its radius of curvature matches that of the substrate.^30,31^

The purpose of the present paper is to show that the combination of capillary condensation and wedge filling leads to a variety of meniscus depinning transitions that occur in a macroscopically long capillary where, at the open end, one of the side walls is slightly longer than the other–see Figure 1. In this case the mismatch means that the meniscus that is formed when the gas condenses to liquid, may be either pinned at different edges of the confining walls, or unpinned, resulting in four possible end meniscus states, e.g., in Figure 1 where the meniscus is pinned at the top edge of slit but is unpinned along the bottom wall, which it meets with the equilibrium contact angle θ, satisfying Young’s equation. As the pressure, or chemical potential, is then further increased toward bulk saturation, the meniscus may then undergo a cascade of depinning and pinning transitions, which depends on the slit width *L*, the overhang distance *D*, and the contact angle θ. We investigate this first macroscopically where we show that the transitions are second-order for partial wetting and third-order for complete wetting. We then show that the different types of meniscus pinned states can be seen in microscopic density functional studies, allowing us to determine a rich phase diagram describing how the capillary condensed liquid phase emerges from the open end as the chemical potential is varied.

**Figure 1:**
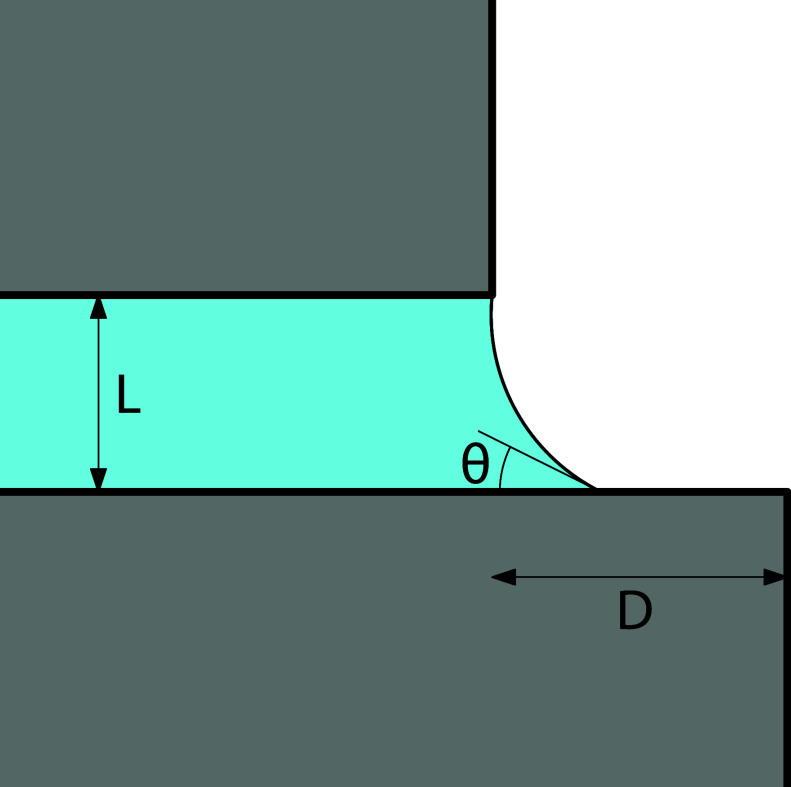
Schematic illustration of a condensed liquid-like phase emerging from the end of an infinitely long capillary slit of width *L* with the overhang *D* due to the extended end of the bottom wall. In the configuration shown here, the meniscus is pinned at the top edge of the capillary but is unpinned at the bottom, where it meets the lower at the equilibrium contact angle θ.

## 2 Macroscopics: Classification of Condensed States

This section outlines the macroscopic characteristics of the condensed states in our model, forming the basis for the analysis of depinning transitions. In the present model, both confining walls are (semi)­infinite. As a result, the phase boundary for capillary condensation coincides with that of an infinite slit and is determined by the chemical potential μ_cc_(*L*), as given by eq 1. The presence of two edges in our geometry introduces the possibility of four distinct condensed morphologies. The meniscus may be attached to both edges (referred to as the **2-state**), to only one edge (either the **1** ^**+**^ **-state** or **1** ^**–**^ **-state**), or to neither edge (the **0-state**); this nomenclature reflects the number and position of the meniscus-pinning edges.

In what follows, we systematically describe the thermodynamic properties of each of these condensed states and examine their relative stability. To this end, we compute the corresponding excess grand potentials (per unit length), measured relative to the reference state illustrated schematically in Figure 2.

**Figure 2:**
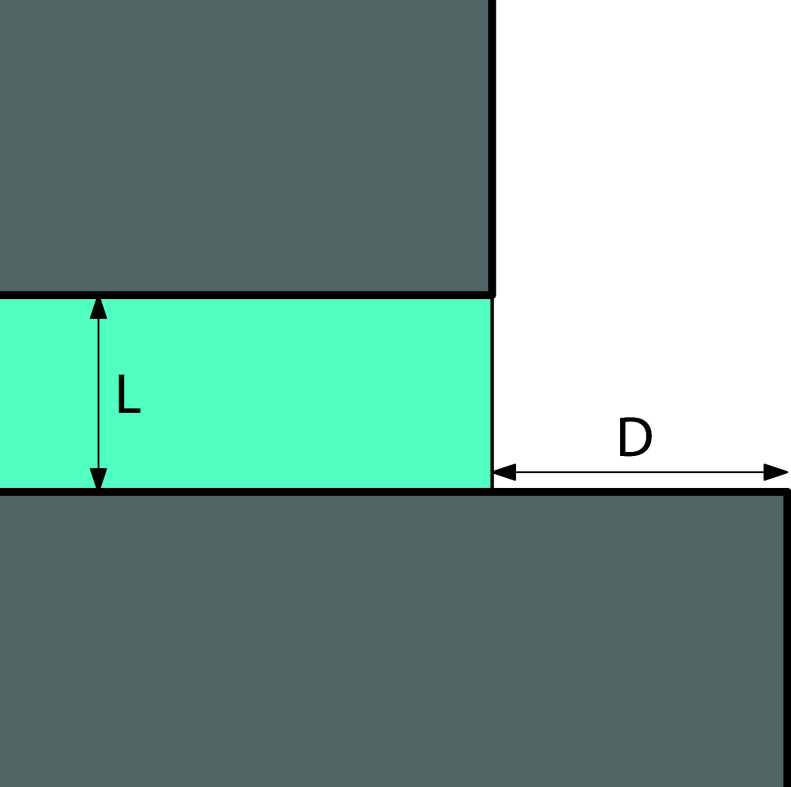
Reference state (a planar projection across the slit) relative to which all the excess grand potentials are determined. The finite free-energy cost due to the liquid–gas interface of the reference system is not considered.

### 2.1 **2**-State

The excess grand potential of the 2-state (i.e., when the meniscus is pinned at both edges, cf. Figure 3) can be written as

$$\Omega_{2}^{ex}=S\Delta p+\gamma l_{m}-\gamma \;D\cos\theta$$ 2

where the terms on the r.h.s. represent, respectively: the excess free energy per unit length due to the presence of metastable liquid of area *S*, the free energy associated with the presence of the meniscus, and the surface free energy gain due to the contact of liquid with the extended portion of the bottom wall.

**Figure 3:**
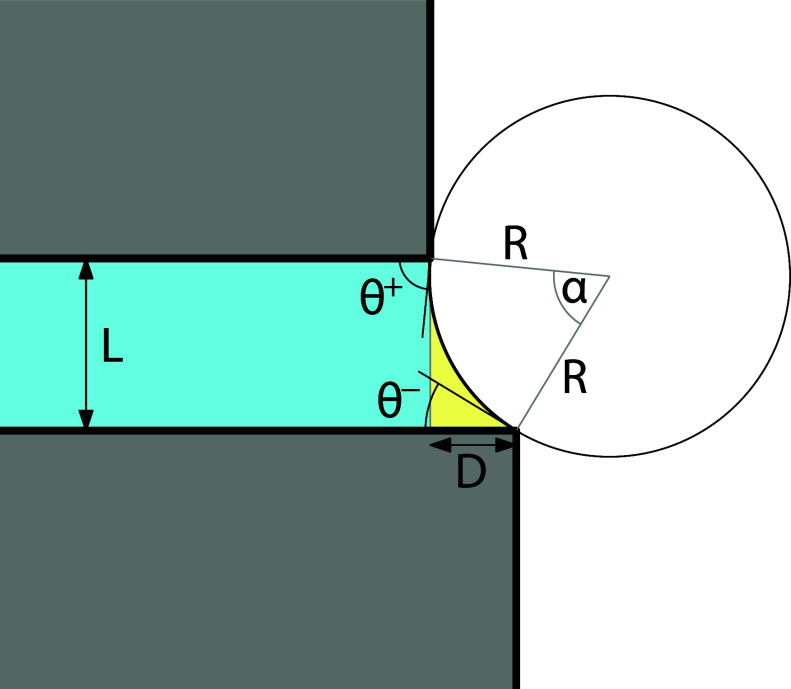
Sketch of the 2-state in which the meniscus is pinned at the upper and the lower edges with corresponding edge contact angles θ^+^ and θ^–^. The meniscus is a section of a circle of radius *R* = γ/Δ*p*.

The area *S* of the liquid region can be expressed as

3 $$S=\frac{LD}{2}+\frac{\sqrt{L^{2}+D^{2}}R\;\cos\frac{\alpha }{2}}{2}-\frac{\alpha }{2}R^{2}$$

where *R* is the radius of curvature of the meniscus and α is the central angle subtended by the meniscus, as shown in Figure 2.

The length of the meniscus is given by

4 $$l_{m}=\alpha R$$

The angle α follows from simple geometry and satisfies

5 $$\sin\frac{\alpha }{2}=\frac{\sqrt{L^{2}+D^{2}}}{2R}$$

Substituting expressions ([Disp-formula eq2]–[Disp-formula eq4]) into eq 1, and using the Laplace relation Δ*p* = γ/*R*, we obtain for the excess grand potential

6 $$\frac{\Omega_{2}^{ex}}{\gamma }=\frac{LD}{2R}+\frac{R}{2}\left(\alpha +\sin\alpha \right)-D\;\cos\theta$$

The central angle α subtended by the meniscus is related to the edge contact angles^32^ θ^+^ and θ^–^ via

7 $$\alpha =\pi -\theta^{+}-\theta^{-}$$

The edge contact angles themselves satisfy the following elementary geometrical conditions

8 $$R\;\cos\theta^{+}+R\;\cos\theta^{-}=L$$

9 $$R\;\sin\theta^{+}-R\;\sin\theta^{-}=D.$$

Combining these with eqs 5 and 7, we obtain a relation for the difference of edge contact angles

10 $$\cos\frac{\theta^{+}-\theta^{-}}{2}=\frac{L}{\sqrt{L^{2}+D^{2}}}$$

or, equivalently

11 $$\sin\frac{\theta^{+}-\theta^{-}}{2}=\frac{D}{\sqrt{L^{2}+D^{2}}}$$

Note that these expressions are independent of the meniscus radius *R*, and thus of the pressure. The difference between the edge contact angles is therefore a purely geometric property.

### 2.2 **1** ^**+**^-State

The excess grand potential of the 1^+^-state is exactly the same as in eq 2 except that *D* is replaced by δ, i.e.

12 $$\frac{\Omega_{1^{+}}^{ex}}{\gamma }=\frac{L\delta }{2R}+\frac{R}{2}\left(\alpha +\sin\alpha \right)-\delta \;\cos\theta$$

where

13 $$\alpha =2\;\arcsin\frac{\sqrt{L^{2}+\delta^{2}}}{2R}$$

The horizontal “overspilling” extension δ is determined by equating the vertical projections of the segment lines of length *R* (see Figure 4) with the slit width, i.e.

14 $$R\;\cos\theta +\sqrt{R^{2}-{\left(\delta +x\right)}^{2}}=L$$

where *x* = *R* sin θ. This leads to

15 $$\delta^{2}-2\delta x+L^{2}-2LR\;\cos\theta =0$$

from which it follows that

16 $$\delta =-R\;\sin\theta +\sqrt{R^{2}\sin^{2}\theta +2LR\;\cos\theta -L^{2}}$$

allowing us to determine the grand potential. We note that the edge contact angle θ^+^ can be determined from the geometrical relation

17 $$R\;\cos\theta^{+}+R\;\cos\theta =L$$

implying

18 $$\cos\theta^{+}=\frac{L}{R}-\cos\theta$$

showing the relation to the Young contact angle.

**Figure 4:**
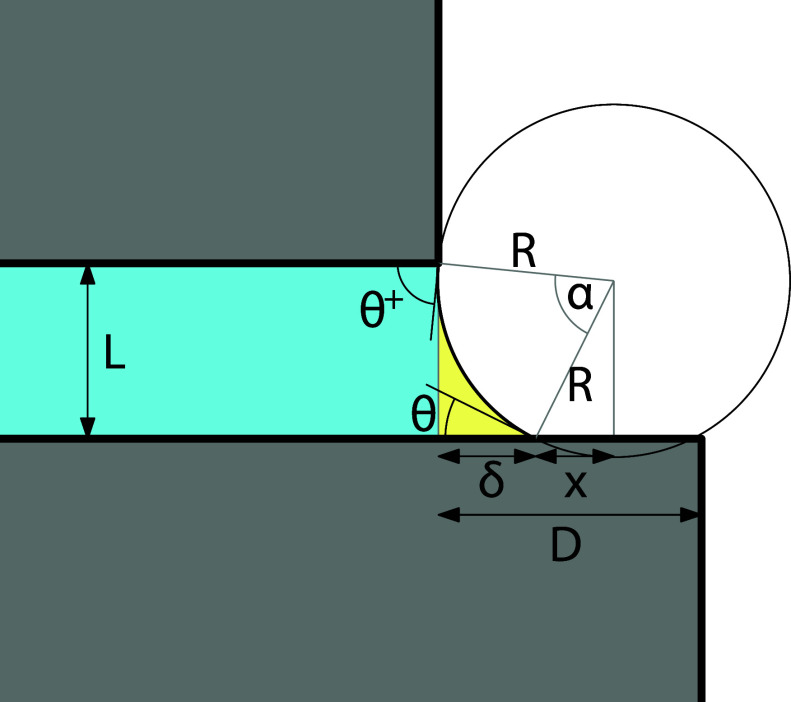
Sketch of the 1^+^-state in which the meniscus is only pinned at the upper edge with the edge contact angles θ^+^ but meets the lower wall at the equilibrium contact angle θ.

### 2.3 **1** ^**–**^-State

The excess grand potential of the 1^–^-state is (see Figure 5)­

19 $$\begin{array}{l} \frac{\Omega_{1^{-}}^{ex}}{\gamma }=\frac{\left(L+\delta \right)D}{2R}+\frac{\alpha }{2}R\\ +\frac{\sqrt{{\left(L+\delta \right)}^{2}+{\;D}^{2}}\cos\frac{\alpha }{2}}{2}-\left(\delta +D\right)\cos\theta . \end{array}$$

Here, δ is determined from

20 $$R^{2}={\left(L+\delta +R\;\sin\theta \right)}^{2}+{\left(R\;\cos\theta -D\right)}^{2}$$

leading to

21 $$\delta^{2}+\left(2L+2R\;\sin\theta \right)\delta +L^{2}+2LR\;\sin\theta +D^{2}-2RD\;\cos\theta =0$$

from which it follows that

22 $$\delta =-L-R\;\sin\theta +\sqrt{R^{2}\sin^{2}\theta +2RD\;\cos\theta -D^{2}}$$

Furthermore, the angle α subtended by the two line segments of length *R* is now

23 $$\alpha =2\;\arcsin\frac{\sqrt{D^{2}+{\;\left(L+\delta \right)}^{2}}}{2R}$$

**Figure 5:**
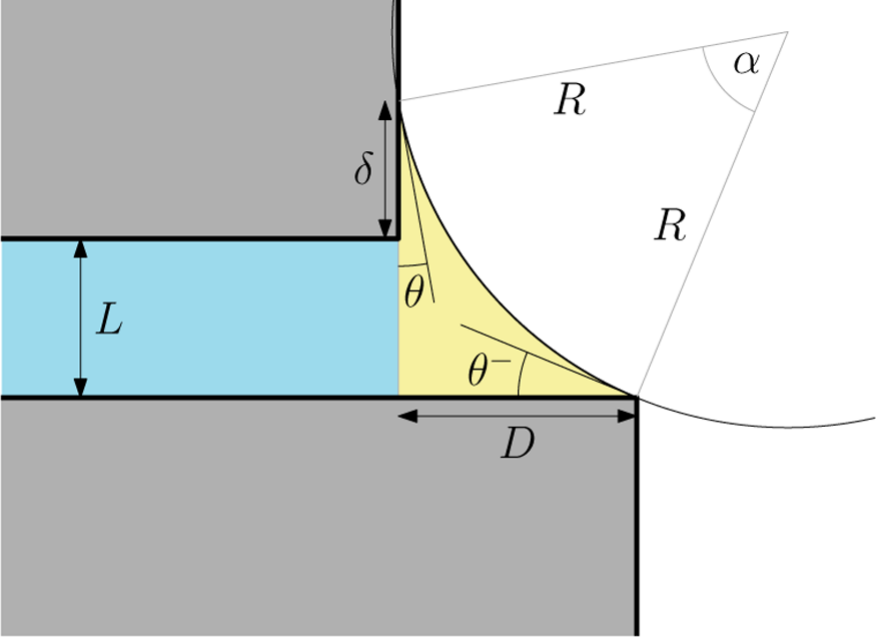
Sketch of the 1^–^-state in which the meniscus is pinned at the lower edge with the edge contact angle θ^–^ but meets the upper wall at the equilibrium contact angle θ.

The angle θ^–^ can be determined from

24 $$R\;\cos\theta^{-}-R\;\sin\theta =L+\delta$$

hence

25 $$\cos\theta^{-}=\sin\theta +\frac{L+\delta }{R}=\frac{\sqrt{R^{2}\sin^{2}\theta +2RD\;\cos\;\theta -D^{2}}}{R}$$

or from

26 $$R\;\cos\theta -R\;\sin\theta^{-}=D$$

implying

27 $$\sin\theta^{-}=\cos\theta -\frac{D}{R}$$

which again shows the modification of the contact angle due to pinning.

### 2.4 **0**-State

The excess grand potential for the 0-state can be written as (see Figure 6)­

28 $$\frac{\Omega_{0}^{ex}}{\gamma }=\frac{\left(L+{\;\delta }^{+}\right)\delta^{-}}{2R}+\frac{R}{2}\left(\alpha +\sin\alpha \right)-\left(\delta^{+}+\delta^{-}\right)\cos\theta$$

**Figure 6:**
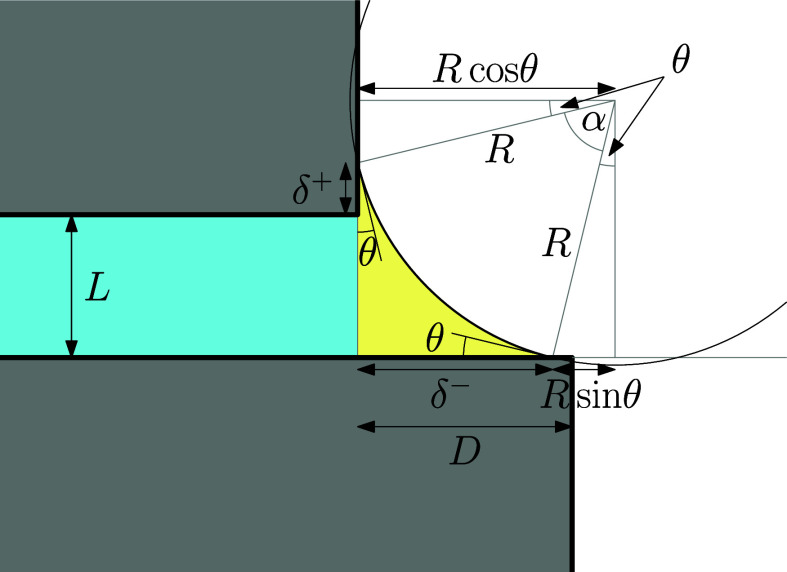
Sketch of the 0-state where the meniscus is unpinned.

The extensions δ^+^ and δ^–^ satisfy

29 $$R\;\cos\theta =L+\delta^{+}+R\;\sin\theta$$

and

30 $$R\;\cos\theta =\delta^{-}+R\;\sin\theta$$

from which it follows that

31 $$\delta^{+}=R\left(\cos\theta -\sin\theta \right)-L$$

and

32 $$\delta^{-}=L+\delta^{+}$$

Similarly, the angle α is given by

33 $$R\;\sin\left(\frac{\alpha }{2}\right)=\frac{\sqrt{{\left(\delta^{+}+L\right)}^{2}+{\delta^{-}}^{2}}}{2}$$

hence using eq 32

34 $$\alpha =2\;\arcsin\frac{\delta^{+}+L}{\sqrt{2}R}$$

Finally, substituting ([Disp-formula eq32]) and ([Disp-formula eq31]) into ([Disp-formula eq28]) we obtain

35 $$\frac{\Omega_{0}^{ex}}{\gamma }=\frac{R{\left(\cos\;\theta -\sin\theta \right)}^{2}}{2}+\frac{R\left(\alpha +\sin\alpha \right)}{2}-\left(2\delta^{+}+L\right)\cos\theta$$

where

36 $$\delta^{+}=R\left(\cos\theta -\sin\theta \right)-L$$

thus specifying the excess grand potential.

## 3 Depinning Transitions

Building on the thermodynamic analysis presented in the previous section, we now determine the specific conditions under which depinning transitions occur between distinct interfacial configurations. These transitions correspond to points where the meniscus becomes unpinned from one geometrical constraint and reattaches to another, leading to abrupt changes in the interfacial morphology. We also characterize the nature of these transitions by examining the derivatives of the excess grand potential Ω^ex^ with respect to the meniscus radius of curvature *R*.

### 3.1 2–1^+^ Depinning

The transition 2 ↔ 1^+^ occurs when δ = *D* (and θ^–^ = θ). This condition defines the radius of curvature of the meniscus at depinning as

37 $$R_{dep}^{2-1^{+}}=\frac{D^{2}+L^{2}}{2L\;\cos\;\theta -2D\;\sin\theta }$$

implying that the transition can occur only if

38 $$\tan\theta <\frac{L}{D}$$

To determine the nature of the transition, we compare the derivatives of the excess grand potentials of the competing states at their coexistence. A simple calculation determines that both derivatives are the same at coexistence, i.e.

39 $$\frac{\partial \Omega_{2}^{ex}}{\partial R}=\frac{\partial \Omega_{1^{+}}^{ex}}{\partial R}$$

However, there is a difference in the second derivatives, which is given by

40 $$\frac{\partial^{2}\Omega_{2}^{ex}}{\partial R^{2}}-\frac{\partial^{2}\Omega_{1^{+}}^{ex}}{\partial R^{2}}=\frac{4\;\sin\theta {\left(L\;\cos\;\theta -D\;\sin\theta \right)}^{4}}{\left(D^{2}+L^{2}\right)\left(D\;\cos\;\theta +L\;\sin\theta \right)\left[2DL\;\cos\theta -\left(D^{2}-L^{2}\right)\sin\theta \right]}$$

which is nonzero for partial wetting, 0 < θ < π, but vanishes in the limit of complete wetting (θ = 0) or complete drying (θ = π). For complete wetting and drying, there is a difference in the value of the third derivatives

41 $$\frac{\partial^{3}\Omega_{2}^{ex}}{\partial R^{3}}-\frac{\partial^{3}\Omega_{1^{+}}^{ex}}{\partial R^{3}}=\frac{4L^{5}}{D^{3}{\left(D^{2}+L^{2}\right)}^{2}},,\left(\theta =0\right)$$

Hence, the 2–1^+^ transition is continuous and, according to the Ehrenfest classification, is of second order for partial wetting and third order for complete wetting.

### 3.2 2–1^–^ Depinning

Analogously, the transition 2 ↔ 1^–^ occurs when δ = 0, giving the corresponding depinning radius

42 $$R_{dep}^{2-1^{-}}=\frac{D^{2}+L^{2}}{2D\;\cos\theta -2L\;\sin\theta }$$

and requiring

43 $$\tan\theta <\frac{D}{L}$$

Similar results apply to this depinning transition. At coexistence, the first derivatives are the same

44 $$\frac{\partial \Omega_{2}^{ex}}{\partial R}=\frac{\partial \Omega_{1^{-}}^{ex}}{\partial R}$$

while for partial wetting the second derivatives are different

45 $$\frac{\partial^{2}\Omega_{2}^{ex}}{\partial R^{2}}-\frac{\partial^{2}\Omega_{1^{-}}^{ex}}{\partial R^{2}}=\frac{4\;\sin\theta {\left(D\;\cos\;\theta -L\;\sin\theta \right)}^{4}}{\left(D^{2}+L^{2}\right)\left(L\;\cos\;\theta +D\;\sin\theta \right)\left[2DL\;\cos\;\theta -\left(L^{2}-D^{2}\right)\sin\theta \right]}$$

This expression vanishes for complete wetting/drying, in which case the difference in the third derivatives is

46 $$\frac{\partial^{3}\Omega_{2}^{ex}}{\partial R^{3}}-\frac{\partial^{3}\Omega_{1^{-}}^{ex}}{\partial R^{3}}=\frac{4D^{5}}{L^{3}{\left(D^{2}+L^{2}\right)}^{2}},,\left(\theta =0\right)$$

showing that the transition is third order.

### 3.3 0–1^+^ Depinning

The transition 0 ↔ 1^+^ corresponds to the vanishing of δ^+^, yielding

47 $$R_{dep}^{0-1^{+}}=\frac{L}{\cos\;\theta -\sin\theta }$$

At the transition

48 $$\frac{\partial \Omega_{1^{+}}^{ex}}{\partial R}=\frac{\partial \Omega_{0}^{ex}}{\partial R}$$

and the difference in the second derivatives takes the form

49 $$\begin{array}{l} \frac{\partial^{2}\Omega_{1^{+}}^{ex}}{\partial R^{2}}-\frac{\partial^{2}\Omega_{0}^{ex}}{\partial R^{2}}=\\ \frac{\left[2+2\;\sin2\theta -\sqrt{1+\sin2\theta }g\left(\theta \right)\right]{\left(\tan\theta -1\right)}^{3}}{4L\sqrt{1+\sin2\theta }} \end{array}$$

where *g*(θ) ≡ 2 cos θ + 3 sin θ + sin3 θ. This is nonzero for partial wetting but vanishes for complete wetting/drying. For complete wetting/drying the difference in the value of the third derivative is given by the simple expression

50 $$\frac{\partial^{3}\Omega_{1^{+}}^{ex}}{\partial R^{3}}-\frac{\partial^{3}\Omega_{0}^{ex}}{\partial R^{3}}=\frac{1}{L^{2}},,\left(\theta =0\right)$$

again showing the transition is of third-order.

### 3.4 0-1^–^ Depinning

Finally, the 0–1^–^ transition occurs when δ^–^ = *D*, which leads to

51 $$R_{dep}^{0-1^{-}}=\frac{D}{\cos\;\theta -\sin\theta }$$

The first derivatives are equal

52 $$\frac{\partial \Omega_{1^{-}}^{ex}}{\partial R}=\frac{\partial \Omega_{0}^{ex}}{\partial R}$$

but the second derivatives satisfy

53 $$\frac{\partial^{2}\Omega_{1^{-}}^{ex}}{\partial R^{2}}-\frac{\partial^{2}\Omega_{0}^{ex}}{\partial R^{2}}=\frac{\sin\theta {\left(\cos\;\theta -\sin\theta \right)}^{3}}{D\;\cos\;\theta }$$

showing the transition is of second order. For complete wetting/drying this reduces to third order transition characterized by

54 $$\frac{\partial^{3}\Omega_{1^{-}}^{ex}}{\partial R^{3}}-\frac{\partial^{3}\Omega_{0}^{ex}}{\partial R^{3}}=\frac{1}{D^{2}},,\left(\theta =0\right)$$

In summary, all depinning transitions share a common feature: they are continuous and second order for partially wetting, but are third order for complete wetting and drying.

## 4 Phase Behavior

The upshot of the results from the previous sections can be summarized as follows. First, since condensation from the gas-like state follows the standard Kelvin eq 1, it is evident that the 1^+^ state first forms at the onset of condensation. As the system approaches saturation–that is, as the pressure deviation δ*p* decreases–other condensed states may emerge. Their appearance depends on: (i) the geometric parameters of the system, specifically the ratio between the extension *D* and the slit width *L*; and (ii) the surface thermodynamic properties, particularly the Young contact angle θ:

$\theta <\frac{\pi }{4}$ : if the contact angle is smaller than the criterion for wedge filling at the right-angle corner, all four condensed states are can occur. However, their emergence further depends on the ratio *D*/*L*.

1. **D < L**: as saturation is approached, the system follows one of the morphological sequences: either 1^+^ → 2 → 1^–^ or simply 1^+^ → 2. The depinning transition 1^+^ → 2 always occurs because tan θ < *L*/*D*. On the other hand, the transition 2 → 1^–^–and hence the existence of the 1^–^ state–is possible only if *D* > D*, where D* = *L* tanθ. The 0-state never arises in this case, as it requires *D* > *L* (along with θ < π/4), as dictated by the symmetry arguments.
2. **D > L**: in this regime, the system always evolves through the sequence 1^+^ → 0 → 1^–^. The 0-state is independent of *D*, and therefore the 1^+^ → 0 transition occurs for any *D* > *L*. As *D* increases, this transition shifts closer to saturation and approaches the 0 → 1^–^ depinning transition. In the limiting case θ = π/4, both phase boundaries coincide with the bulk condensation. The 2-state cannot occur here, as it fails to meet the condition tan θ < *L*/*D* required to develop from the 1^+^ state.

The stability of the admissible capillary states in this regime is summarized in Table 1.

**I tbl1:** $\theta <\frac{\pi }{4}$

(a) *D* < *L*		(b) *D* > *L*	
**state**	pressure interval	**state**	pressure interval
gas	$R<\frac{L\;\sec\theta }{2}$	gas	$R<\frac{L\;\sec\theta }{2}$
1^+^	$\frac{L\;\sec\theta }{2}<R<\frac{L^{2}+D^{2}}{2L\;\cos\theta -2D\;\sin\theta }$	1^+^	$\frac{L\;\sec\theta }{2}<R<\frac{L}{\cos\theta -\sin\theta }$
2	$\frac{L^{2}+D^{2}}{2L\;\cos\theta -2D\;\sin\theta }<R<\frac{L^{2}+D^{2}}{2D\;\cos\theta -2L\;\sin\theta }$	0	$\frac{L}{\cos\theta -\sin\theta }<R<\frac{D}{\cos\theta -\sin\theta }$
1^–^	$\frac{L^{2}+D^{2}}{2D\;\cos\theta -2L\;\sin\theta }<R$	1^–^	$\frac{D}{\cos\theta -\sin\theta }<R$

$\theta >\frac{\pi }{4}$ : when the contact angle is larger than the criterion for wedge filling at the right-angle corner, the phase behavior is significantly simpler.

1. **D < L**: the system evolves from the gas phase into the 1^+^ state, and then possibly to the 2-state, provided that the condition tanθ < *L*/*D* is met. If this condition is not satisfied, the system remains in the 1^+^ state up to saturation. The 1^–^ state cannot form in this case, as the upper wall cannot accommodate a meniscus at the Young contact angle.
2. **D > L**: in this regime, the 0-state is excluded, and consequently, the 1^–^ state cannot arise since it cannot evolve from the 1^+^ state. Therefore, 1^+^ is the only possible condensed state.

These results are illustrated in Figure 7 for several contact angles.

**Figure 7:**
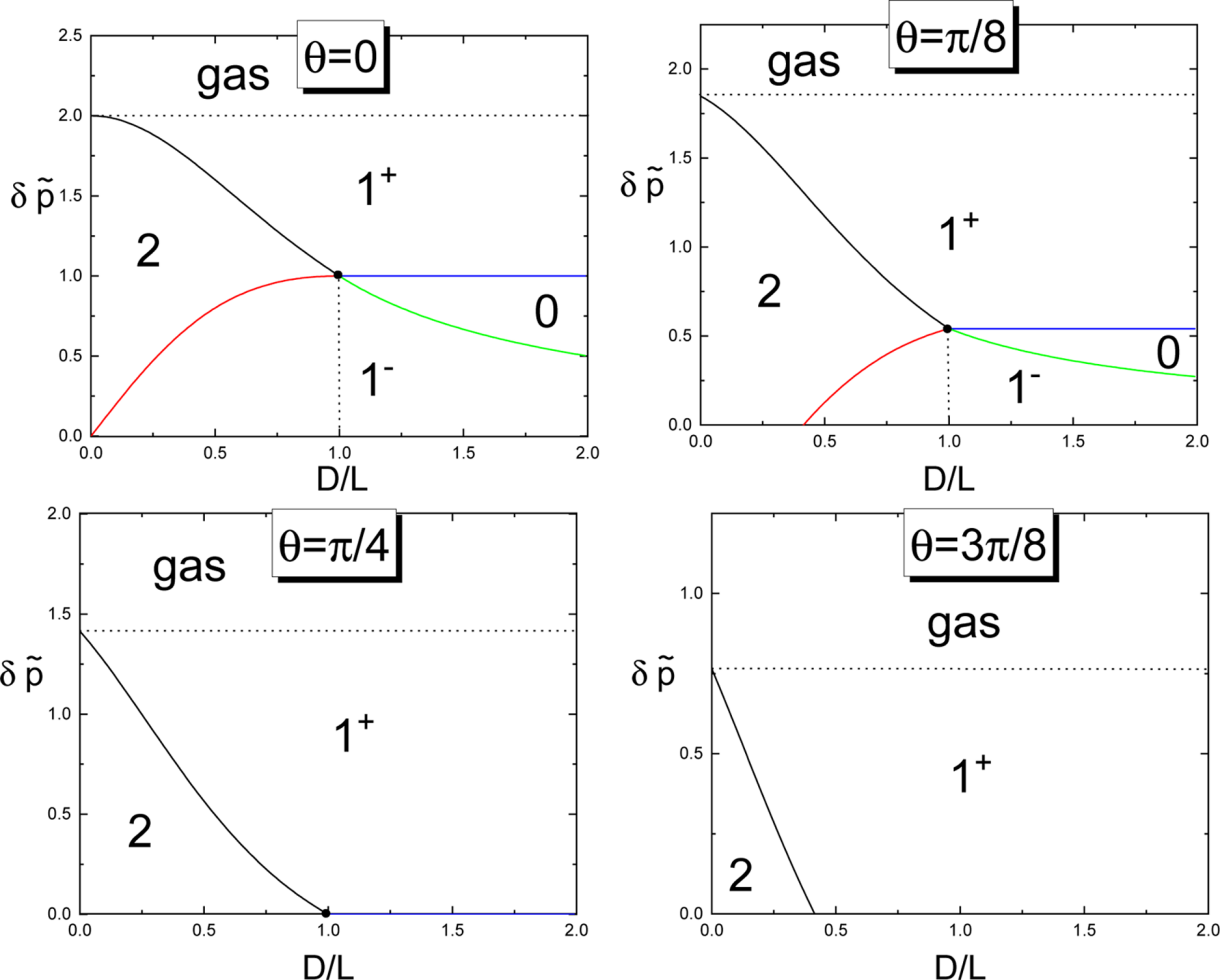
Phase diagrams illustrating the phase boundaries between different condensed states for various values of the contact angle. The solid lines denote the boundaries between the following phases: 2–1^+^ (black), 2–1^–^ (red), 0–1^+^ (blue) and 0–1^–^ (green). The horizontal dashed line indicates the capillary–condensation transition. The axes are scaled using the dimensionless variables δp̃ = L/R and *D*/*L*.

## 5 Microscopic Model

In this section, we present a microscopic model to test our macroscopic predictions for depinning transitions at the edges of semi-infinite slits. To this end, we employ classical density functional theory (DFT), where the central quantity is the one-body fluid density, or density profile, ρ­(**r**). Within DFT, the equilibrium density profile, ρ_eq_(**r**), is the one which minimizes the grand potential functional Ω­[ρ] ^12,33^

55 Ω[ρ]=F[ρ]+∫drρ(r)[V(r)−μ]

where *V*(**r**) is the external potential, and F[ρ] is the intrinsic Helmholtz free-energy functional that encapsulates all fluid–fluid interactions. Although generic models applicable for a wide range of systems have been developed, it is more common to tailor the approximation of the intrinsic free energy functional to the specific microscopic model defined by the fluid–fluid potential. For simple fluids, reliable perturbative approaches have been developed in the spirit of the early van der Waals theory. This technique is based on a separation of the fluid–fluid pairwise potential, *u*(*r*) (where *r* is the distance between the centers of the two interacting particles), into a purely repulsive part, *u*_rep_(*r*), which primarily determines the fluid structure, and an attractive part, *u*_att_(*r*), treated as a perturbation.

In this work, we model fluid–fluid interactions using the truncated, nonshifted Lennard-Jones (LJ) potential. The attractive component is given by

56 $$u_{att}\left(r\right)=\{\begin{array}{ll} 0; & r<\sigma ,\\ -4\varepsilon {\left(\frac{\sigma }{r}\right)}^{6}; & \sigma <r<r_{c},\\ 0; & r>r_{c}. \end{array}$$

with a cutoff distance *r*_c_ = 2.5, σ. The harsh repulsion at short distances is mapped onto a hard-sphere fluid of diameter σ, which serves as a natural reference system for both homogeneous and inhomogeneous fluid states.^34^

The external potential *V*(**r**) accounts for the presence of the confining walls, schematically illustrated in Figure 1. In this work, we consider two distinct wall models. The first corresponds to purely repulsive hard walls. In the second, the walls are modeled as being composed of LJ atoms uniformly distributed with number density ρ_*w*_. Each wall atom exerts a long-ranged (nontruncated) LJ potential

57 $$\phi \left(r\right)=4\varepsilon_{w}\left[{\left(\frac{\sigma_{w}}{r}\right)}^{12}-{\left(\frac{\sigma_{w}}{r}\right)}^{6}\right]$$

where ε_*w*_ and σ_*w*_ characterize the interaction strength and length scale, respectively. The net potential arising from both walls is obtained by integrating eq 57 over the respective wall volumes. We begin by evaluating the contribution from the bottom wall, which extends horizontally up to a distance *D* from the origin of the Cartesian coordinate system. Its potential is given by

58 $$\begin{array}{l} V^{\left(b\right)}\left(x,z\right)=\rho_{w}\int_{-\infty }^{D}dx^{\prime }\int_{-\infty }^{\infty }dy^{\prime }\int_{-\infty }^{0}dz^{\prime }\\ \times \phi \left(\sqrt{{\left(x-x^{\prime }\right)}^{2}+y^{\prime 2}+{\left(z-z^{\prime }\right)}^{2}}\right), \end{array}$$

which be decomposed into its repulsive and attractive contributions

59 $$V^{\left(b\right)}\left(x,z;D\right)=V_{12}^{\left(b\right)}\left(x,z;D\right)+V_{6}^{\left(b\right)}\left(x,z;D\right)$$

The repulsive part of the LJ wall potential is given by

60 $$\begin{array}{l} V_{12}^{\left(b\right)}\left(x,z;D\right)=\pi \varepsilon_{w}\sigma_{w}^{12}\rho_{w}[\psi_{12}\left(x-D,z\right)\\ -\psi_{12}\left(\infty ,z\right)-\psi_{12}\left(x-D,\infty \right)] \end{array}$$

where the auxiliary function ψ_12_(*x*, *z*) reads

61 $$\begin{array}{l} \psi_{12}\left(x,z\right)=-[128\left(x^{16}+z^{16}\right)+448\left(x^{14}z^{2}+z^{14}x^{2}\right)\\ +560\left(x^{12}z^{4}+z^{12}x^{4}\right)+280\left(x^{10}z^{6}+z^{10}x^{6}\right)\\ +35x^{8}z^{8}]/\left[2880z^{9}x^{9}{\left(x^{2}+z^{2}\right)}^{7/2}\right] \end{array}$$

with its asymptotic behavior given by

62 $$\psi_{12}\left(x,\infty \right)=-\frac{2}{45}\frac{1}{x^{9}},\psi_{12}\left(\infty ,z\right)=-\frac{2}{45}\frac{1}{z^{9}}$$

The attractive part of the potential is similarly expressed as

63 $$\begin{array}{l} V_{6}^{\left(b\right)}\left(x,z;D\right)=-\frac{\pi }{3}\varepsilon_{w}\sigma_{w}^{6}\rho_{w}[\psi_{6}\left(x-D,z\right)\\ -\psi_{6}\left(x-D,\infty \right)-\psi_{6}\left(\infty ,z\right)] \end{array}$$

with

64 $$\psi_{6}\left(x,z\right)=-\frac{2x^{4}+x^{2}z^{2}+2z^{4}}{2z^{3}x^{3}\sqrt{x^{2}+z^{2}}}$$

and the asymptotic limits

65 $$\psi_{6}\left(x,\infty \right)=-\frac{1}{x^{3}},\psi_{6}\left(\infty ,z\right)=-\frac{1}{z^{3}}$$

Finally, the potential due to the top wall *V* ^(*t*)^(*x*, *z*) is obtained by symmetry and takes the form

66 $$V^{\left(t\right)}\left(x,z\right)=V^{\left(b\right)}\left(x,L-z;0\right)$$

where the coordinate transformation reflects the vertical inversion relative to the slit height *L*.

Continuing within the perturbative framework for the intrinsic free-energy functional, we decompose it into the ideal-gas contribution and an excess term

67 $$F\left[\rho \right]=F_{id}\left[\rho \right]+F_{ex}\left[\rho \right]$$

where the ideal-gas part is known exactly

68 $$F_{id}\left[\rho \right]=\beta^{-1}\int dr\rho \left(r\right)\left[\ln\left(\rho \left(r\right)\Lambda^{3}\right)-1\right]$$

with Λ denoting the thermal de Broglie wavelength and β = 1/*k*_*B*_ *T* representing the inverse temperature.

The excess part, $F_{ex}\left[\rho \right]$ , is further separated into a hard-sphere contribution and an attractive part, with the latter treated in a mean-field approximation

69 $$F_{ex}\left[\rho \right]=F_{hs}\left[\rho \right]+\frac{1}{2}\iint drdr^{\prime }\rho \left(r\right)\rho \left(r^{\prime }\right)u_{att}\left(|r-r^{\prime }|\right)$$

The hard-sphere contribution, $F_{hs}\left[\rho \right]$ , is approximated using Rosenfeld’s fundamental measure theory (FMT),^35^ which provides an accurate description of short-range correlations

70 $$F_{hs}\left[\rho \right]=\frac{1}{\beta }\int dr\Phi \left(\{n_{\alpha }\}\right)$$

where Φ is the excess free energy density, depending on a set of weighted densities *n*_α_. In the original Rosenfeld formulation, these consist of four scalar and two vector weighted densities, each obtained via a convolution of the density profile ρ­(**r**) with a corresponding weight function

71 $$n_{\alpha }\left(r\right)=\int dr^{\prime }\rho \left(r^{\prime }\right)w_{\alpha }\left(r-r^{\prime }\right)\alpha =\left\{0,1,2,3,v1,v2\right\}$$

where

72 $$\begin{array}{llll} \omega_{3}\left(r\right) & =\Theta \left(R-r\right), & \omega_{2}\left(r\right) & =\delta \left(R-r\right)\\ \omega_{1}\left(r\right) & =\omega_{2}\left(r\right)/4\pi R, & \omega_{0}\left(r\right) & =\omega_{2}\left(r\right)/4\pi R^{2}\\ \omega_{v2}\left(r\right) & =\frac{r}{R}\delta \left(R-r\right), & \omega_{v1}\left(r\right) & =\omega_{v2}\left(r\right)/4\pi R. \end{array}$$

Here, Θ is the Heaviside step function, δ is the Dirac delta function, and R=σ/2 is the hard-sphere radius.

Minimizing the grand potential functional (55) yields the Euler–Lagrange equation

73 $$V\left(r\right)+\frac{\delta F_{hs}}{\delta \rho_{eq}\left(r\right)}+\int dr^{\prime }\rho \left(r^{\prime }\right)u_{att}\left(|r-r^{\prime }|\right)=\mu$$

which can be recast into a self-consistent expression for the equilibrium density profile

74 $$\rho_{eq}\left(r\right)=\Lambda^{-3}\;\exp\left[\beta \mu -\beta V\left(r\right)+c^{\left(1\right)}\left(r\right)\right]$$

here, *c* ^(1)^(**r**) = *c*_hs_^(1)^(**r**)+*c*_att_^(1)^(**r**) is the one-body direct correlation function, composed of two parts: the hard-sphere contribution, which is given by

75 $$c_{hs}^{\left(1\right)}\left(r\right)=-\sum_{\alpha }\int dr^{\prime }\frac{\partial \Phi \left(\{n_{\alpha }\}\right)}{\partial n_{\alpha }}w_{\alpha }\left(r^{\prime }-r\right)$$

and the attractive part, which is

76 $$c_{att}^{\left(1\right)}\left(r\right)=-\beta \int dr^{\prime }u_{att}\left(|r-r^{\prime }|\right)\rho_{eq}\left(r^{\prime }\right)$$

Equation 74 was solved numerically using Picard’s iteration on a two-dimensional rectangular grid with a spatial resolution of 0.1 σ. The convolution integrals appearing in eqs 71, 75, and 76 were computed efficiently using the fast Fourier transform (for implementation details, see ref 36). By choosing the wall parameters as σ_*w*_ = σ and ε_*w*_ = ε, the wetting temperature of the attractive walls is *T*_*w*_/*T*_*c*_ ≈ 0.8, where the bulk critical temperature is *k*_*B*_ *T*_*c*_/ε ≈ 1.41.^37^

## 6 Numerical Results

In this section, we present our numerical DFT results for two slit models: (a) a repulsive system formed by hard walls and (b) an attractive system formed by Lennard-Jones (LJ) walls. Following the approach described in the previous section, we determined sequences of equilibrium density profiles by varying the bulk density (chemical potential) and constructed the corresponding phase diagrams to compare with theoretical predictions. We considered two temperatures, *k*_B_ *T*/ε = 1.1 and 1.3, i.e., one below and one above the wetting temperature of the LJ wall, and three geometries with aspect ratios *D*/*L* = 0.7, 1.3, and 2.0, fixing *L* = 10 σ.

We use the following criteria to determine if a configuration is pinned at the top and bottom. We begin with the slightly easier case of complete wetting (θ = 0) or complete drying (θ = π). First, we draw the meniscus contour, where the density is midway between the bulk gas and liquid. A configuration is deemed pinned at the top corner if the contour has an overhang and unpinned if it has not. The depinning is then equivalent to the condition that the fitted edge contact angle at the upper wall is θ^+^ = π/2, which is just the condition that the meniscus meets the vertical wall tangentially. To determine the pinning at the bottom wall, we fit the meniscus to a circle and determine its radius and center. Here, we must distinguish between two cases, transitions 0 → 1^–^ and 2 → 1^–^. In state 0, the circle center should lie on the diagonal line which emerges at an angle of 45° from the point of vertical projection of the upper wall onto the lower wall. When the center is below the diagonal, the meniscus is deemed pinned at the lower wall (state 1^–^). Similarly, in state 2, the circle center should lie on the line which is normal to the straight line which connects the corners and passes through its midpoint. Again, when the center is below the axis, the meniscus is considered depinned from the upper wall (state 1^–^).

Very similar criteria are applied to the case of partial wetting. Indeed, for the 0 and 2 states they are identical. For the 1^+^ and 1^–^ states the direction of the diagonal along which the center must lie is simply shifted by the value of the contact angle with respect to the lower wall and upper wall, respectively.

### 6.1 Hard Walls: Complete Drying

We begin with the simple case of purely repulsive hard walls corresponding to a contact angle θ = π, independent of temperature. Consequently, the results obtained at both studied temperatures are nearly identical and thus we only present the representative density profiles for *k*_*B*_ *T*/ε = 1.3 (Figure 8), which also illustrate the procedure used to distinguish between the different morphological states. We note that, unlike the sketches shown in Figures 1–6, the roles of liquid and gas are now interchanged. Macroscopically, the phase behavior of this system is expected to be equivalent to that of completely wet walls (θ = 0).

**Figure 8:**
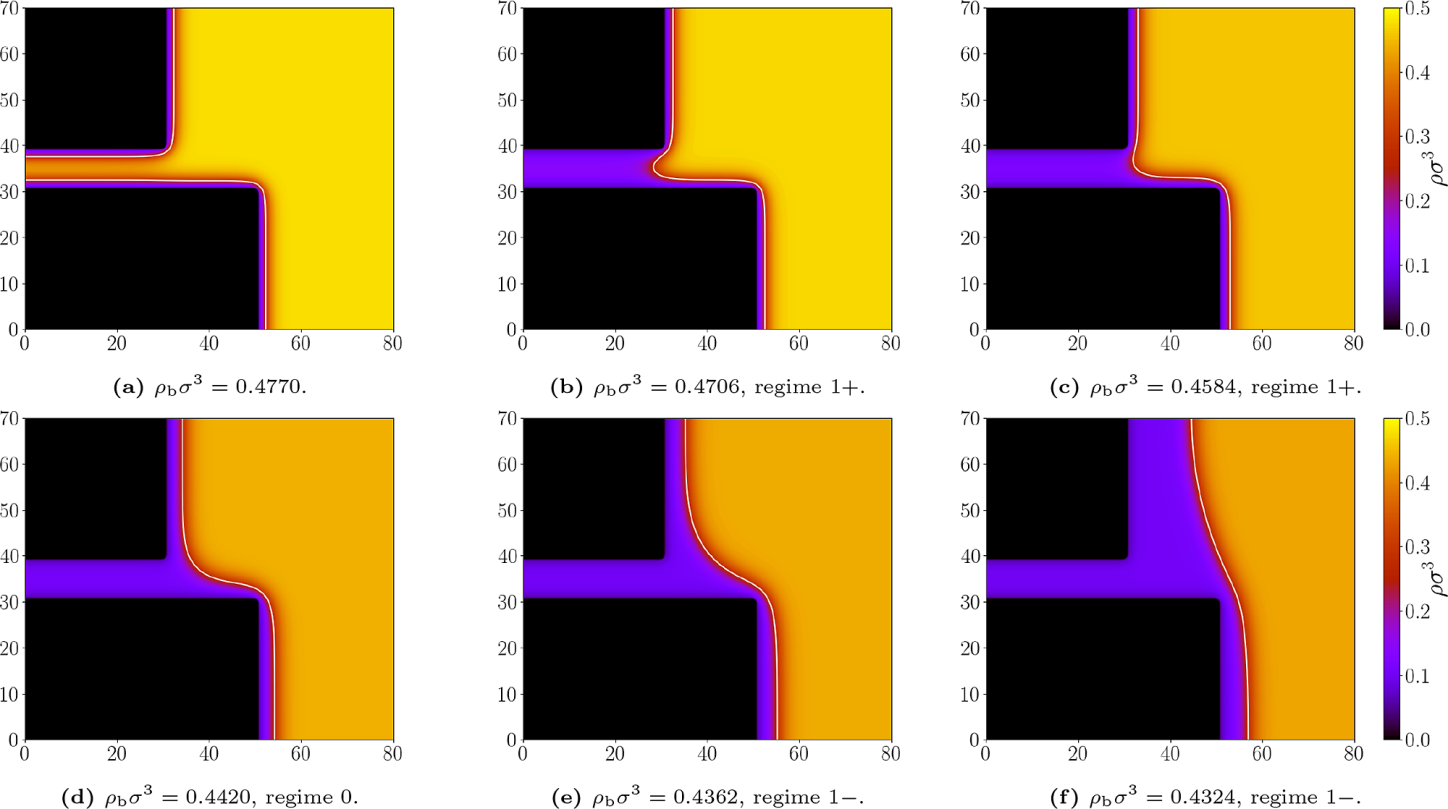
Representative density profiles for the geometry *L* = 10σ and *D* = 20σ; and bulk densities ρ_b_σ^3^: (a) 0.4770, (b) 0.4706, (c) 0.4584, (d) 0.4420, (e) 0.4362, (f) 0.4324. Repulsive walls, temperature *k*_*B*_ *T*/ε = 1.3. The white line marks the liquid–gas interface at which ρ = (ρ_*l*_ + ρ_*g*_)/2. Appropriate (de)­pinning regimes are labeled.

Far from saturation, the slit is completely filled with liquid (see Figure 8a), meaning that we are still above the capillary transition. Slightly below the capillary evaporation point, the slit becomes filled with gas, and the system adopts the 1^+^ state, where the meniscus is pinned at the upper corner (Figure 8b). The meniscus depins from the upper corner once it no longer turns inside the slit before curving outward to meet the lower wall. For *D*/*L* > 1, this leads to the depinned (0) state (see Figure 8d). Further flattening of the meniscus produces the 1^–^ regime, when the circle center departs from the diagonal and the meniscus becomes pinned to the lower corner (Figure 8e). As saturation is approached, the meniscus becomes progressively flatter while remaining pinned at the lower corner, i.e., in the 1^–^ state (Figure 8f).

Next, we consider the case *D*/*L* < 1. In contrast to the previous results, the depinned (0) state is now absent, consistent with theoretical predictions. Instead, and again as expected, the system forms the 2-state, with the meniscus pinned at both corners. To locate the 1^+^ → 2 transition, we examined whether the meniscus approached the line connecting both wall corners perpendicularly. The 2 → 1^–^ transition occurs when the meniscus depins from the upper corner, i.e., when it no longer turns around the upper corner inside the slit before curving outward to meet the lower corner. Upon further approach to saturation, the meniscus flattens while remaining pinned at the lower edge. The full sequence of states is displayed in Figure 9.

**Figure 9:**
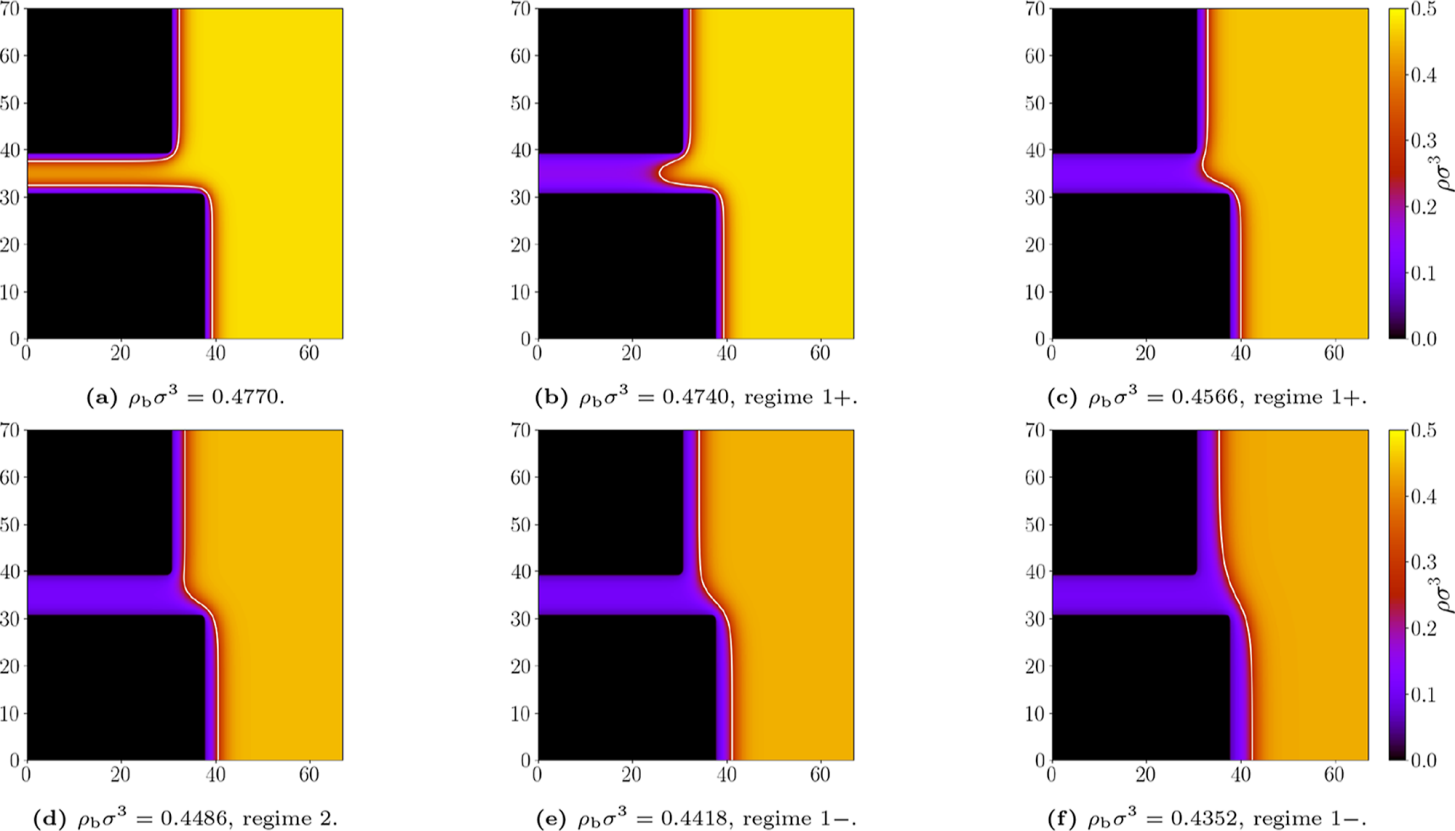
Representative density profiles for the geometry *L* = 10σ and *D* = 7σ; and bulk densities ρ_b_σ^3^: (a) 0.4770, (b) 0.4740, (c) 0.4566, (d) 0.4486, (e) 0.4418, (f) 0.4352. Repulsive walls, temperature *k*_*B*_ *T*/ε = 1.3. The white line marks the liquid–gas interface at which ρ = (ρ_*l*_ + ρ_*g*_)/2. Appropriate (de)­pinning regimes are labeled.

By identifying the bulk densities corresponding to each regime, we constructed the phase diagram shown in Figure 10, which is in excellent agreement with the macroscopic predictions.

**Figure 10:**
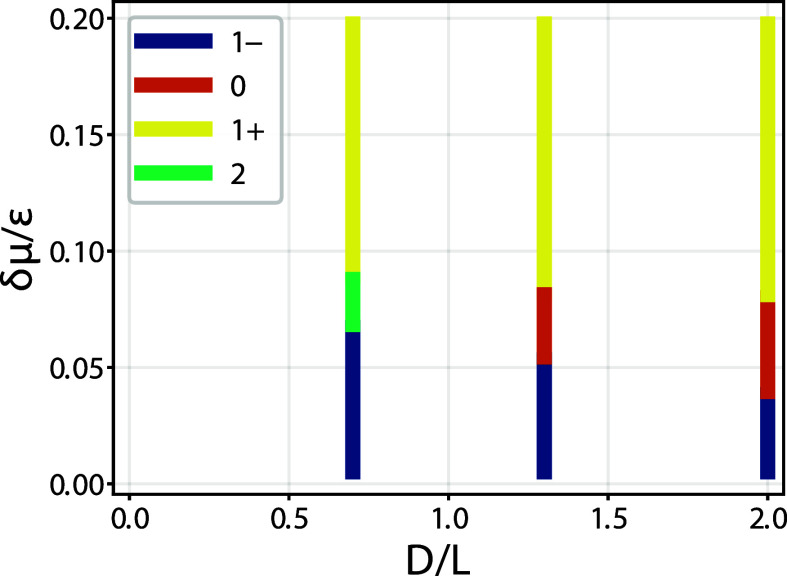
Phase diagram for complete drying as obtained from DFT for a model of hard walls. For overhang ratio *D*/*L* > 1 the condensed phase changes from a 1^+^ state to a 1^–^ state via a 0 state, involving two depinning transitions as the deviation from the bulk saturation δμ is decreased. However, when *D*/*L* < 1 the change from a 1^+^ state to a 1^–^ occurs via double pinned 2 state. These features are in good qualitative agreement with the macroscopic diagram shown as the top-left diagram. For *k*_*B*_ *T*/ε = 1.3.

### 6.2 Attractive Walls: Complete Wetting

We now turn to the model with attractive walls and examine the complete wetting regime (*k*_B_ *T*/ε = 1.3). From a macroscopic perspective, one expects a scenario analogous to that of hard walls, with the roles of the liquid and gas phases reversed. However, it is instructive to compare the two cases to assess the influence of packing effects, which are significantly stronger in the present case.

Figure 11 shows a sequence of density profiles for the geometry with the largest overlap, *D* = 20σ. At low bulk densities, only thin adsorbed layers are formed (see Figure 11a). At the onset of capillary condensation, the system adopts the 1^+^ state (Figure 11b). As the bulk density increases, the meniscus remains in the 1^+^ regime while the adsorbed layers grow thicker (Figure 11c). Over a narrow density interval, the meniscus detaches from the upper corner and slides upward while remaining free along the lower wall, forming the depinned (0) state (Figure 11d). A configuration close to the 0 → 1^–^ transition is shown in Figure 11e, which we identify as belonging to the 1^–^ regime. Near saturation, the meniscus becomes nearly flat and firmly pinned at the lower corner (Figure 11f).

**Figure 11:**
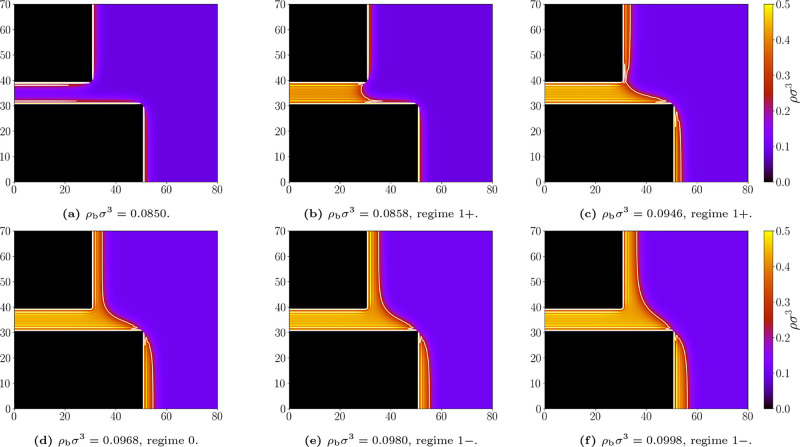
Representative density profiles for the geometry *L* = 10σ and *D* = 20σ; and bulk densities ρ_b_σ^3^: (a) 0.0850, (b) 0.0858, (c) 0.0946, (d) 0.0968, (e) 0.0980, (f) 0.0998. Attractive walls, temperature *k*_*B*_ *T*/ε = 1.3. The white line marks the liquid–gas interface at which ρ = (ρ_*l*_ + ρ_*g*_)/2. Appropriate (de)­pinning regimes are labeled.

For *D*/*L* = 1.3 (see Figure 12), the situation differs qualitatively. No 0-state is detected; instead, the system undergoes a 1^+^ → 2 transition, as expected for narrower slits (*D*/*L* < 1). This contrasts with the drying case for the same geometry (cf. Figure 10). The difference clearly arises from the thick wetting films at the walls, which effectively reduce the slit width. Consequently, the phase behavior for *D*/*L* = 1.3 closely resembles that for *D*/*L* = 0.7. The resulting phase diagram for all considered parameters is shown in Figure 13.

**Figure 12:**
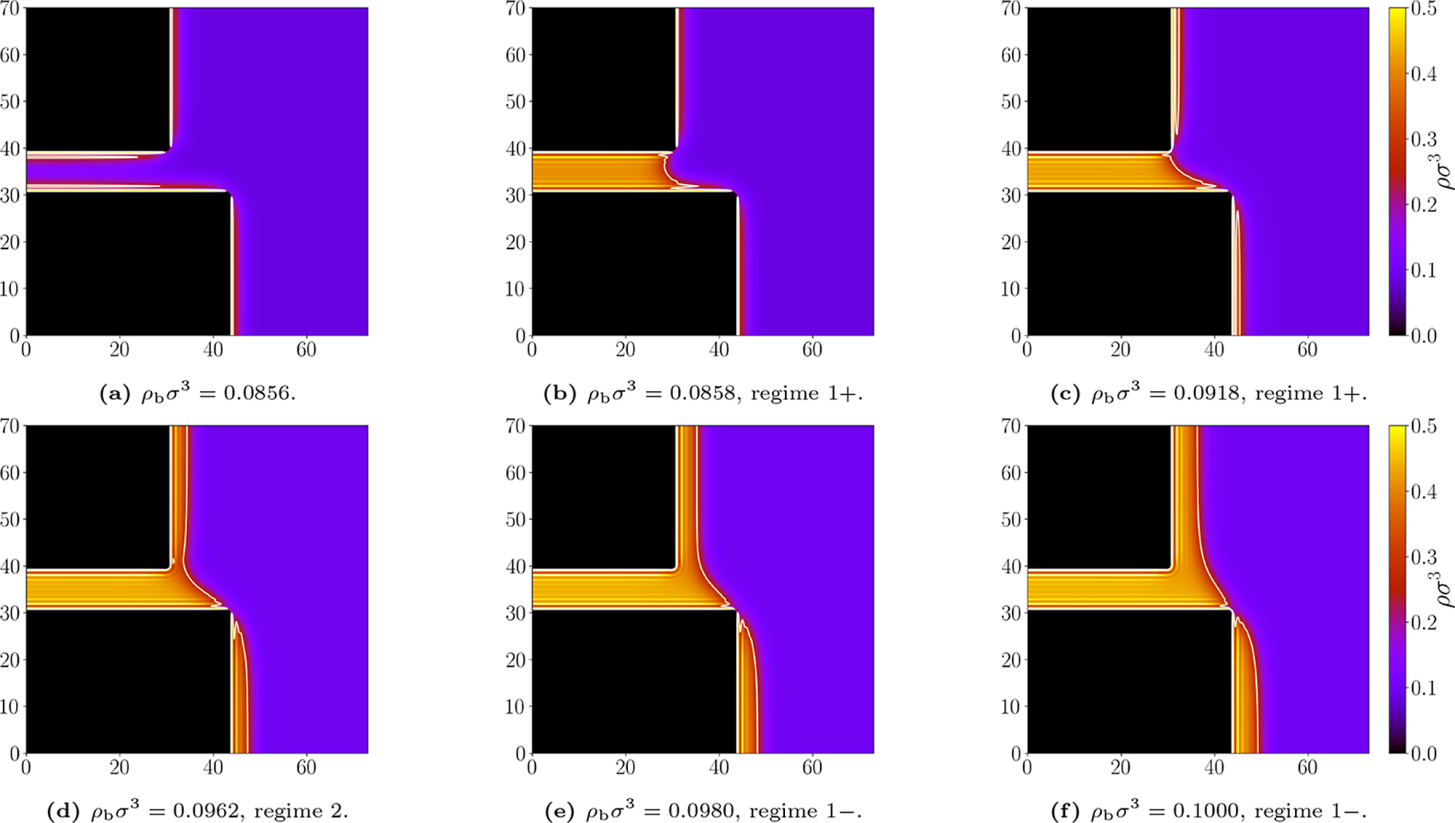
Representative density profiles for the geometry *L* = 10σ and *D* = 13σ; and bulk densities ρ_b_σ^3^: (a) 0.0856, (b) 0.0858, (c) 0.0918, (d) 0.0962, (e) 0.0980, (f) 0.1000. Attractive walls, temperature *k*_*B*_ *T*/ε = 1.3. The white line marks the liquid–gas interface at which ρ = (ρ_*l*_ + ρ_*g*_)/2. Appropriate (de)­pinning regimes are labeled.

**Figure 13:**
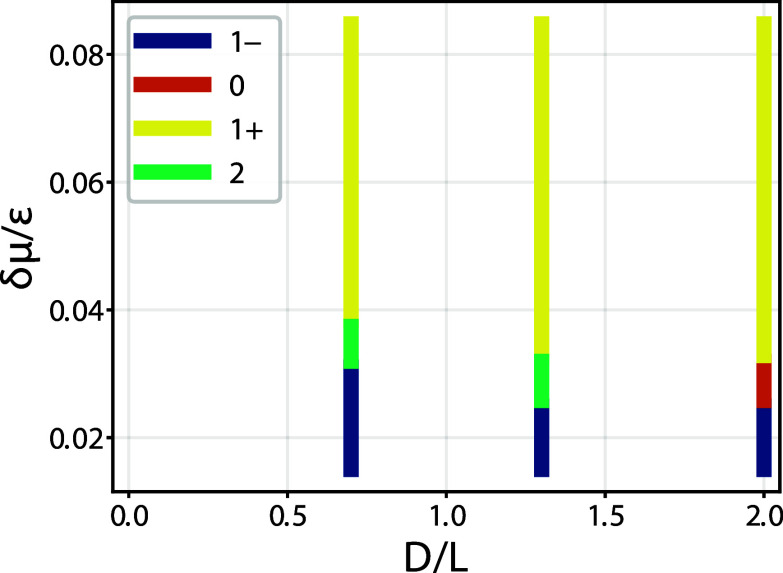
Phase diagram for completely wet walls obtained from DFT for a model with attractive Lennard-Jones walls at temperature *k*_*B*_ *T*/ε = 1.3. For sufficiently large overhang ratios *D*/*L*, the condensed phase changes from a 1^+^ state to a 1^–^ state via an intermediate 0 state, involving two depinning transitions as the deviation from the bulk saturation δμ is decreased. For lower values of the ratio *D*/*L*, the change from a 1^+^ state to a 1^–^ occurs via double pinned 2 state. This behavior is consistent with the macroscopic phase diagram shown in the top-left panel However, the DFT results indicate that the boundary between these two regimes occurs at *D*/*L* > 1, deviating somewhat from the macroscopic prediction–which neglects strong packing effects near the wall–that anticipates this change at *D*/*L* = 1.

### 6.3 Attractive Walls: Partial Wetting

When *k*_*B*_ *T*/ε = 1.1 the confining walls are partially wet with contact angle θ ≈ 35°. There is only a microscopic adsorption on the walls and no reduction on the effective slit width is expected. For *D*/*L* = 2 the system behaves again according to the macroscopic predictions evolving from through 1^+^, 0 and 1^–^ states (see Figure 15). Note that the interval of states corresponding to the 1^–^ states is rather narrow, as expected. Similar behavior is also observed for *D*/*L* = 1.3. However, for the smallest ratio, *D*/*L* = 0.7, the condensed system remains largely in the 1 + state only turning to the 2-state close to saturation (see Figure 16). This is again in line with our macroscopic expectations (cf. Figure 7). The phase diagram displaying the equilibrium phases for the three ratios for partially wet walls is in Figure 14.

**Figure 14:**
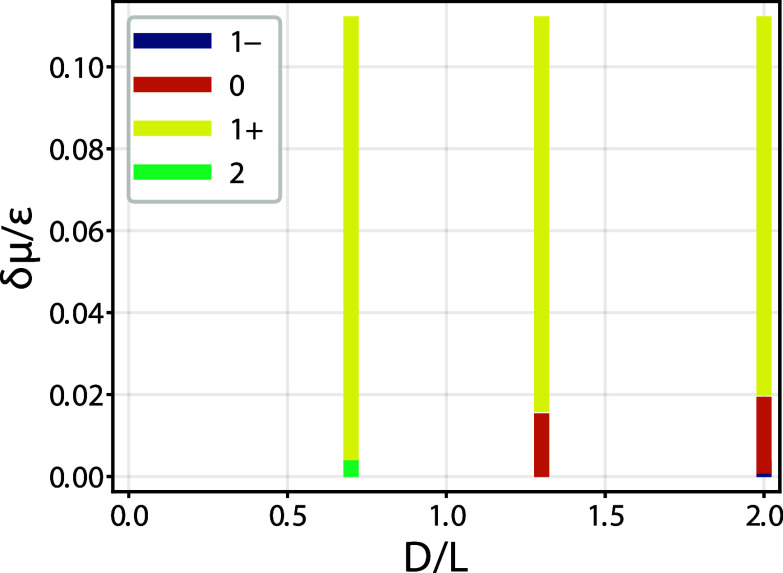
Phase diagram for partially wet walls obtained from DFT for a model with attractive Lennard-Jones walls at temperature *k*_*B*_ *T*/ε = 1.1. For sufficiently large overhang ratios *D*/*L*, the condensed phase undergoes a transition from a 1^+^ state to a 1^–^ state via an intermediate 0 state, although the regions corresponding to the 0 and 1^–^ states are very narrow (cf. Figure 15). For smaller values of *D*/*L*, the condensed phase changes from the 1^+^ state to a double pinned 2 state, involving only a single (de)­pinning transition. These results are in full qualitative agreement with the macroscopic predictions shown in Figure 7 (see the top-right and bottom-left panels).

**Figure 15:**
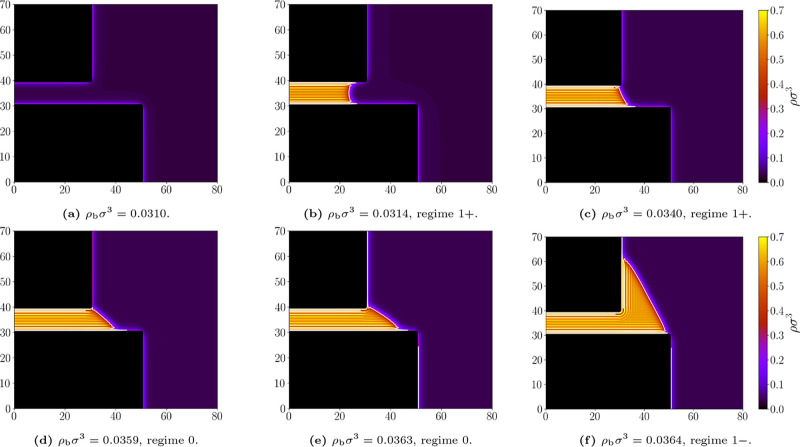
Representative density profiles for the geometry *L* = 10σ and *D* = 20σ; and bulk densities ρ_b_σ^3^: (a) 0.0310, (b) 0.0314, (c) 0.0340, (d) 0.0359, (e) 0.0363, (f) 0.0364. Attractive walls, temperature *k*_*B*_ *T*/ε = 1.1. The white line marks the liquid–gas interface at which ρ = (ρ_*l*_ + ρ_*g*_)/2. Appropriate (de)­pinning regimes are labeled.

**Figure 16:**
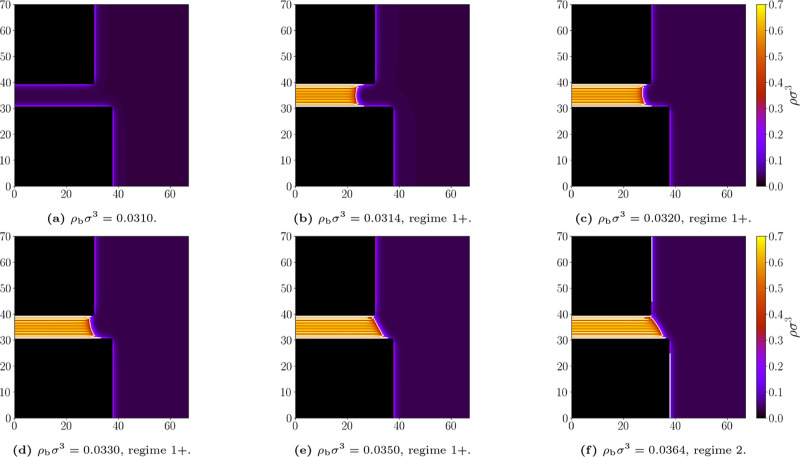
Representative density profiles for the geometry *L* = 10σ and *D* = 7σ; and bulk densities ρ_b_σ^3^: (a) 0.0310, (b) 0.0314, (c) 0.0320, (d) 0.0330, (e) 0.0350, (f) 0.0364. Attractive walls, temperature *k*_*B*_ *T*/ε = 1.1. The white line marks the liquid–gas interface at which ρ = (ρ_*l*_ + ρ_*g*_)/2. Appropriate (de)­pinning regimes are labeled.

### 6.4 Comparison of Drying and Wetting Scenarios

Finally, we compare complete wetting and complete drying. While these phenomena have identical macroscopic descriptions, with the roles of gas and liquid are reversed, they are markedly different at the microscopic, molecular level. The condensed phase exhibits stronger molecular correlations and layering in contact with the wall. Consequently, the meniscus for complete wetting has a more pronounced molecular structure than for complete drying. These differences are illustrated in Figure 17, which shows four pairs of density profiles for wetting and drying at corresponding values of δμ.

**Figure 17:**
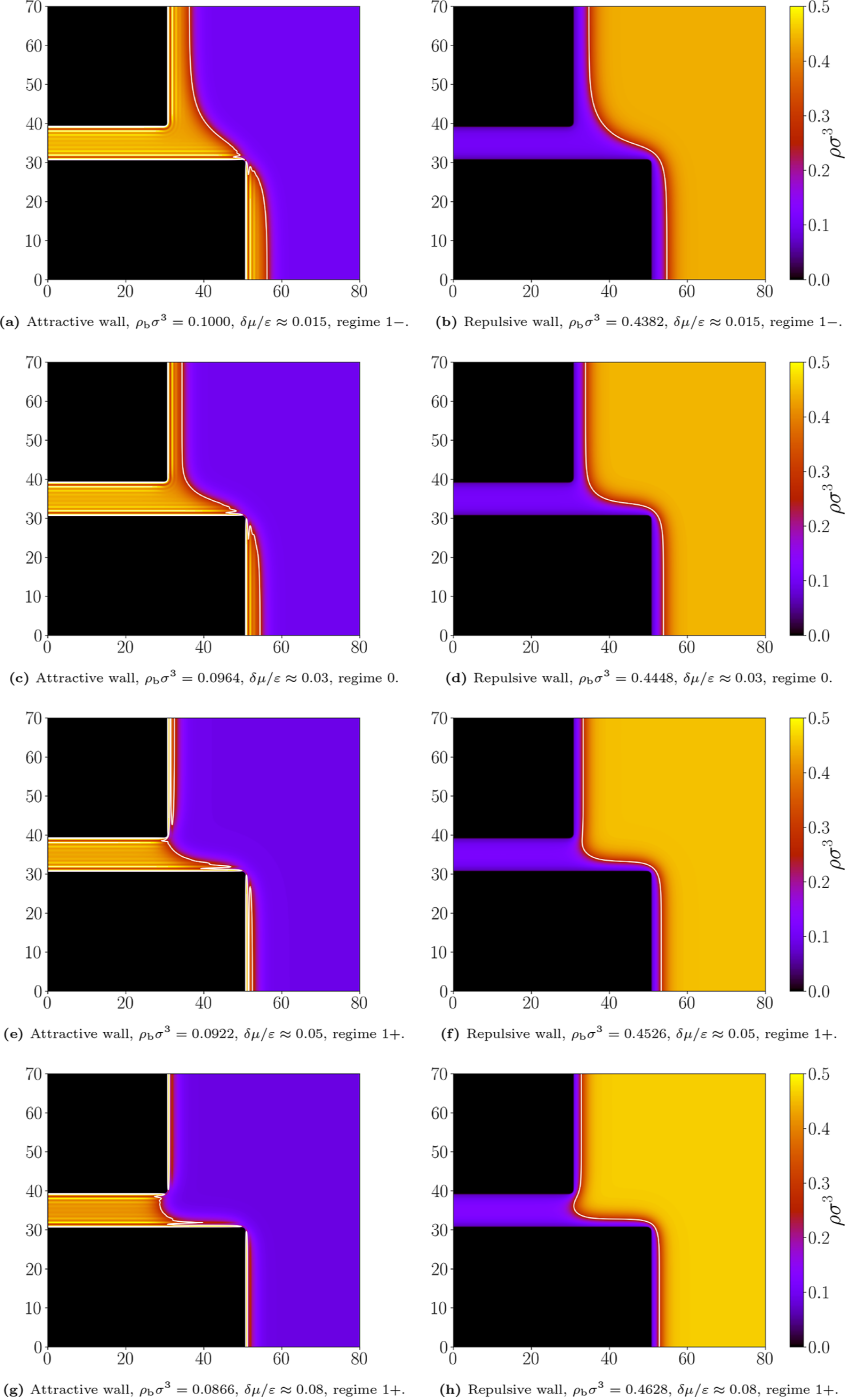
Equilibrium density profiles for the slit with *L* = 10σ and *D* = 20σ at a temperature *k*_*B*_ *T*/ε = 1.3. Comparison of the effect of attractive and repulsive walls for the corresponding δμ/ε: (a) and (b) 0.015; (c) and (d) 0.03; (e) and (f) 0.05; (g) and (h) 0.08. The white line marks the liquid–gas interface at which ρ­(**r**) = (ρ_*l*_ + ρ_*g*_)/2. Bulk densities and pertinent regimes are indicated.

## 7 Conclusion

In this work, we have presented a comprehensive macroscopic and microscopic description of depinning transitions in confined fluids. The occurrence of depinning transitions, distinguishing among various capillary states, arises from the influence of sharp confining-wall edges, which are ubiquitous in real systems. By combining geometric (macroscopic) analysis with classical density functional theory (DFT), we established the criteria governing the stability and transformation of meniscus morphologies at the edges of semi-infinite slits. The macroscopic theory predicts four distinct condensed states-fully pinned (2), partially pinned (1^+^ and 1^–^), and depinned (0)–whose existence and mutual transitions depend sensitively on the wall separation ratio *D*/*L* and the Young contact angle θ. The analytic results reveal that the depinning transitions are continuous, being of second order for partial wetting and of third order for complete wetting.

Microscopic DFT calculations confirmed these predictions with high accuracy. The numerical results reproduce the full sequence of morphological transitions anticipated by the macroscopic model and delineate the corresponding phase boundaries in terms of the chemical potential deviation δμ and the geometric aspect ratio. The computed density profiles clearly illustrate how the meniscus evolves on approaching saturation and how these transformations depend on the wetting properties of the confining walls.

While qualitatively consistent with the macroscopic predictions, the microscopic results also reveal important finite-size effects that round otherwise sharp transitions and modify the accessible range of morphologies. In particular, the formation of thick adsorbed films under complete wetting conditions effectively narrows the slit, thereby suppressing the depinned regime even for *D*/*L* > 1. This demonstrates the subtle interplay between microscopic interfacial structure and macroscopic boundary constraints.

In summary, by introducing the concept of the edge contact angle within a semi-infinite slit geometry, we have elucidated the role of sharp edges in determining the phase behavior of confined fluids. We identified all possible condensed morphologies induced by the presence of edges, located the corresponding pinning–depinning transitions, determined their order, and constructed phase diagrams for various contact angles. The theoretical predictions were supported by microscopic DFT calculations. Future work may extend these insights to cylindrical or chemically heterogeneous geometries, where walls with different contact angles could suppress or stabilize certain states. Further progress will also require a detailed microscopic analysis of the near-edge region and its coupling to layering transitions. Finally, the macroscopic framework can be refined by incorporating for the presence of wetting layers and also the effects of net microscopic forces responsible for transition rounding. These topics are the subject of ongoing investigation.
